# Supplementary material for: Study the muti-bolt fastening under different load positions in gear rack drilling rig
Source: PLoS One. 2023 Aug 31;18(8):e0290427. doi: 10.1371/journal.pone.0290427 (PMC10470948; doi:10.1371/journal.pone.0290427)
Supplement: Attachment — Submitted filename: Response to Reviewers.docx [file pone.0290427.s002.docx]

Reviewer #1:

**1. The paper is weird that the model validation is after the parametric analysis.**

In order to make the paper structure more logical, the model validation section has been modified after the paper modeling calculation. The model validation part is placed in section 3.2 of the paper, after the Finite element setting and before the finite element result.

**2. section 4.3 is too short to be a seperate section.**

Due to the adjustment of the structure of the paper, the analysis results of the original section 4.3 have been placed in the model verification section.

**3. I do not believe the authors know how to write an academic paper, please refer to: https://doi.org/10.1016/j.jcsr.2018.12.020. You will find some useful information.**

The reference "https://doi.org/10.1016/j.jcsr.2018.12.020." has provided some writing ideas for my paper in terms of the structure of the paper and research philosophy, and has been rewritten and cited for the structure of the paper with the citation number "24".

**4. The** **matierial propertis, boundary conditions,** **interaction properties should be introduced in details.**

The finite element pre-processing settings such as element type settings, material properties, contact settings and boundary are described in sections 3.11 and 3.12.

**5. The paper needs a major revision for sure, not only the content, but also the organization and language.**

Thank you for your valuable and thoughtful comments. We have carefully checked and improved the English writing in the revised manuscript.

**6. The quality of the figures also needs improvement.**

The quality of the paper's pictures has been enhanced, particularly in terms of the structural form of the object under study, the composition of its parts, and the additional description of boundary conditions for calculations.

Reviewer #2:

**1. It is written in rather poor English. It is full of editorial errors.**

Thank you for your valuable and thoughtful comments. We have carefully checked and improved the English writing in the revised manuscript.

**2. Bibliographic descriptions are missing.**

The bibliographic section has been added.

**3. The authors use erroneous citations to items from the literature list.**

The paper has carefully checked the problem of reference citations, and the problem of multiple citations for the same paper has been corrected.

**4. No explanatory drawing of the adopted case study.**

In the section on parameter learning, the thesis illustrates the parameters and their numerical values with this figure10 and word explanation.

**5. Unintelligible Figures 4 and 7 (Which bolts are being referred to? What is their position?).**

The bolts and load positions are explained both in words and graphically, as shown in Figures 4 and 7.

**6. Incomplete description of experimental studies.**

The overall structural framework of the paper has been revised, and the original experimental analysis section has been modified to the validation of the finite element model, enhancing the description of the original experimental results.

Reviewer #3: This paper presents a study on multi-bolt transmission system of the drilling rigs. The topic is of great interests from both academic and industry. And the paper is well-written and therefore suggested to be considered to be published, with the following comments addressed:

**1. Could the authors provide more details on the comparisons results of tests and numerical and theoretical predictions? It's critical to show the accuracy of the proposed predicting methods.**

The Theoretical Calculations, Numerical Simulations and Strain Tests section provides further research details and explanations of boundary conditions, material properties and contact settings. The details are added in section 2.3 case study, section 3.11 Element and Material properties, section 3.12Interaction properties and Boundary conditions, 3.2 model validation.

**2. In the description to numerical modelling, could the authors please accordingly cite the references for the procedures that referring to some existing and well-known models in literature?**

For the creation of finite element model in numerical simulation, well-known model and literature are used in the simulation of relevant setting basis and evaluation method. The relevant references have been cited in the paper, numbered 25, 28 and 30.

**3. It's more logic to validate the developed numerical model first, then used it to conduct parametric studies. So, it's suggested to switch the order of sections in the current paper.**

Thank you for your comments and improvement measures on my paper. The overall structural framework of the paper has been revised, and the original experimental analysis section has been modified to the validation of the finite element model.

**4. There are a few more relevant literature published recently. The authors may refer to them to get the updated ideas on the behaviour of high strength steel multi-bolt connections' failure in different failure modes, like staggered fracture, bearing and block tearing:**

[1] Jiang, K., Zhao, O., Tan, KH. (2020). Experimental and numerical study of S700 high strength steel double shear bolted connections in tension. Engineering Structures, 225, 111175.

[2] Jiang, K., Tan, KH., Zhao, O. (2021). Net section fracture of S700 high strength steel staggered bolted connections. Thin-Walled Structures,164, 107904.

[3] Jiang, K., Tan, KH., Zhao, O., Gardner, L. (2021). Block tearing of S700 high strength steel bolted connections: Testing, numerical modelling and design. Engineering Structures, 246, 112979.

Thanks for the excellent reference work. Combined with reply '2', several references have been added to the Methods section of the Simulation Model section.

Reviewer #4: The paper proposes a finite element simulation method for gear-rack drilling rigs which present several bolted joints. Some aspects regarding the modeling description and the validation of the results need to be improved. Therefore, the revised version of the manuscript should consider the following amendments:

**1. Section 1 – For greater clarity, report the expanded names for the acronyms ECCS and CSCS.**

Check the explanation of all the first abbreviations in the paper, and complete the missing cases. The original abbreviations ECCS and CSCS were deleted, and the two Standards were changed to Eurocodes and Standards of China.

**2. Figure 2 – Report in this figure all the names of the components mentioned in Section 2.1 and give a closer view of the fasteners to help identify their position and details.**

The schematic diagram of the rack fastened on the derrick has been included in the revised structure diagram presented in Figure 2. Additionally, the names of the individual components have been added for clarity.

**3. Figure 3 is not recalled in the text.**

The manuscript has undergone the necessary revisions and updates. Figure 3 now showcases a 5-bolt spring model and specifies two distinct load positions within the figure.

**4. Eq. (5) – Explain the evaluation of Exx and Eyy. The method described in Refs. [17,24] is aimed at the analysis of bolts connecting composite plates, hence the plates present different Young’s Modulus along the x and y directions. Is in this case necessary to distinguish two different Young’s Moduli?**

As per your professional suggestion, appropriate changes have been made accordingly. The manuscript has been modified about the issue of Young’s Modulus, that being the focus of this study is not on composite material modification. The changes are as follows: Exx Eyy is merged into E.

**5. Section 2.4 – The distinction between load positions 1 and 2 is not clear. Describe more this aspect and add a figure to support the description.**

Modified. Loading position 1 and loading position 2 are visually illustrated in the following part through picture 3.

**6. Section 3.2 – Give further details about the differential deformation between rack and bolt. How is it measured and what are the technical implications?**

Specific details explaining the location of data measurement and specific data are added in 3.3 Finite Element Result.

**7. Section 3.4 – It would be interesting to add a parametric study regarding the preload of the bolts.**

Added in section 4.5. In fact, the effect of the preload on the effect of the rack is significant.

**8.Is it possible to compare some results of Section 3 with the analytical approach to give validation of the numerical method?**

The effect of the Z-direction is ignored in the theoretical model developed in this paper because it only considers the two-dimensional displacement of the deformation gauge in the X- and Y-directions. Considering the reliability of the finite element model, the accuracy of the finite element model is verified by experiments.

**9.The introduction should discuss other references about the FE analysis of composite bolted joints:**

<https://doi.org/10.1016/j.compositesb.2021.109378>

<https://doi.org/10.1016/j.compstruct.2020.112166>

https://doi.org/10.1016/j.compositesb.2019.05.034

https://doi.org/10.1016/j.compstruct.2020.112770

<https://doi.org/10.1016/j.compstruct.2020.113199>

<https://doi.org/10.1016/j.prostr.2020.02.078>

In response to the references provided, inspired by the structure of the thesis and the way it was written, it has been cited, with the citation numbers being: 16 29 27 12 15 14 respectively.

**10. The English of the manuscript needs to be carefully checked to correct misspells, typos. Some sentences also need to be rephrased.**

For the full text writing, abbreviations and spelling have been meticulously reviewed and rectified. The paper has undergone substantial enhancements, with particular emphasis on refining the language and improving the clarity of arguments.
