# Supplementary material for: Study the muti-bolt fastening under different load positions in gear rack drilling rig
Source: PLoS One. 2023 Aug 31;18(8):e0290427. doi: 10.1371/journal.pone.0290427 (PMC10470948; doi:10.1371/journal.pone.0290427)
Supplement: Attachment — Submitted filename: Response to Reviewers.docx [file pone.0290427.s003.docx]

Reviewer #2:

The article looks much better, but still needs improvement. However, already in terms of minor changes, which include:

**1. Remove the 'Content' section.**

The 'content' section has been deleted.

2. **Use correct citations to items included in the literature list (for example, 'Su et al. [7]' instead of 'Su [7]' in line 56). This must be checked for all citations. Look at other articles on how this should be done, such as these: https://doi.org/10.3390/lubricants10050075 or https://doi.org/10.3390/ma15124354.** **References must exactly match the items given in the literature list. The citation in line 82, for example, is incorrect; instead of 'McCarthy[17]' it should be 'Sharos and McCarthy [17]'. Check the others.**

The reference citation format and the literature review author citation have been modified according to the reference (https://doi.org/10.3390/lubricants10050075) format.

**3. The description for [13] in the literature list is incomplete. Check the others.**

Incomplete references and problems with matches in the literature list have been corrected, as detailed in the paper's references.

**4. Correct the mathematical notations in lines 121 to 123. What exactly does i(i=e,1-4) mean?**

Thank you for improving the quality of the paper. The original symbolic representation is prone to ambiguity, and the formulae have been re-corrected and refined in Figure 3.

**5. Inform the reader what the bolt numbers in Figures 4 and 9 mean. Which bolts do these numbers refer to?**

In order to better illustrate the meaning of the bolt numbers, the position of the individual bolts has been supplemented in Figures 3 and 6.

**6. Extend the description of the FEM model. In what way were the bolts preloaded?**

Yes, the additions have been made to the original description in order to make the finite element model set-up description more detailed. Among them, regarding the loading method of the bolt preloading force, the expansion is completed and modified as follows: 'The effect of preloading the bolt is achieved by applying a relative clamping force of 469 kN to the bolt and nut.'”
